# Differences in metagenome coverage may confound abundance-based and diversity conclusions and how to deal with them

**DOI:** 10.1093/ismeco/ycaf140

**Published:** 2025-09-10

**Authors:** Borja Aldeguer-Riquelme, Luis M Rodriguez-R, Konstantinos T Konstantinidis

**Affiliations:** School of Civil & Environmental Engineering and School of Biological Sciences, Georgia Institute of Technology, Atlanta, GA 30332, United States; Department of Biomedicine and Dentistry, European University of Andalucia, Málaga, 29010, Spain; Department of Microbiology & Digital Science Center (DiSC), University of Innsbruck, Innsbruck, A-6020, Austria; School of Civil & Environmental Engineering and School of Biological Sciences, Georgia Institute of Technology, Atlanta, GA 30332, United States

**Keywords:** metagenome, coverage, differential abundance, diversity, nonpareil

## Abstract

The importance of rarefying ecological or amplicon sequencing data to a standardized level of diversity coverage for reliable diversity comparisons across samples is well recognized. However, the importance of diversity coverage, i.e. the fraction of the genomic diversity of a sample sequenced, in comparative shotgun metagenomic studies remains frequently overlooked. Using both *in silico* and natural metagenomes from a wide range of environments, we demonstrate that uneven metagenome coverage can result in misleading biological conclusions, particularly for identifying differentially abundant features, i.e. groups of genes or genomes assigned to the same protein family or taxonomic rank, respectively, and for comparing diversity between samples. The main underlying cause is that not all members of a feature may be detectable, and thus counted, across such unevenly covered metagenomes depending on the sequencing effort applied and the underlying member-abundance curves. Unfortunately, 99.5% of previous comparative metagenomic studies have overlooked this metric, suggesting that their reported results might be misleading. We show that achieving high Nonpareil coverage (≥0.9), a metric that estimates metagenome diversity coverage, is the most reliable strategy to mitigate this issue. When high Nonpareil coverage is not achievable, such as for highly diverse and complex samples like soils, we show that standardizing (or subsampling) metagenomic datasets to the same Nonpareil coverage, rather than sequencing effort, prior to comparative analysis provides for more accurate results. We provide a set of practical recommendations and the corresponding Python scripts to help researchers to assess and standardize metagenome diversity coverage for their comparative analyses.

## Introduction

Modern microbiome studies frequently employ sequencing approaches, such as amplicon sequencing or shotgun metagenomics, to answer ecological-, evolutionary-, or diversity-related questions. However, only a fraction of the whole microbial community is typically sequenced in these studies. Therefore, determining the amount of diversity recovered by the sequencing effort applied (hereinafter, diversity coverage) is essential for understanding the representativeness of the dataset and the reliability of the conclusions obtained from it. That is, significant deviations in the diversity coverage between datasets might distort the inferred differences between the corresponding communities in terms of their phylogenetic or functional diversity [1]. Strategies to estimate the diversity coverage of a dataset usually rely on a redundancy metric or the detection of single occurrences (singletons) of diversity units, such as Operational Taxonomic Units (OTUs) or reads. These methods provide both qualitative (e.g. rarefaction curves) [2] and quantitative estimates (e.g. Turing–Good’s coverage) [3] of diversity coverage.

Traditional rarefying analysis attempts to account for (or standardize) uneven diversity coverage between samples by subsampling all datasets to the same number of sequences or/and diversity of features (e.g. % of total OTUs). Rarefying has been shown to be necessary when comparing 16S rRNA gene amplicon datasets of varied diversity coverage or number of sequences in order to infer differentially abundant taxa, differences in diversity and more between the datasets [4–6]. This standardization strategy has been widely adopted in the literature due to its implementation in popular tools for analyzing 16S rRNA gene amplicon data, such as Mothur or QIIME2 [7, 8]. Moreover, it is now well recognized that rarefying amplicons by sequencing effort (i.e. number of sequences used) is an insufficient, and often counter-productive method [1, 9], and diversity coverage standardization should be instead used [10–12]. There are several reasons supporting this claim. First, samples standardized by size may have different degrees of diversity coverage and thus, representativeness, depending on their species richness and species-abundance distributions. In a simplified example, a sample with 1,000 species will require around 10X more sequencing effort to be as well covered as a sample with only 100 species and the same degree of evenness. Second, standardizing samples by size does not satisfy the replication principle while diversity coverage-based standardization does [10, 11]. The replication principle ensures that the ratio of diversity richness between samples is maintained when comparing their subsampled datasets [10]. Thus, by preserving this principle, the diversity coverage-based standardization approach better reflects the real differences in diversity between samples or their subsamples than the size-based standardization. Third, diversity coverage-based projections are finite because this variable is bound between 0 and 1, unlike size-based projections. This is important because it allows computing asymptotic estimates of diversity and (some) closed-form confidence intervals [13]. Despite the recognized importance of diversity coverage in ecological and amplicon studies, this metric is still largely ignored in comparative shotgun metagenomic studies. This is presumably because it is technically much more challenging to estimate diversity coverage with (largely) non-overlapping sequences like in shotgun metagenomic datasets (or simply metagenomes) relative to overlapping amplicon data.

**Figure 1:**
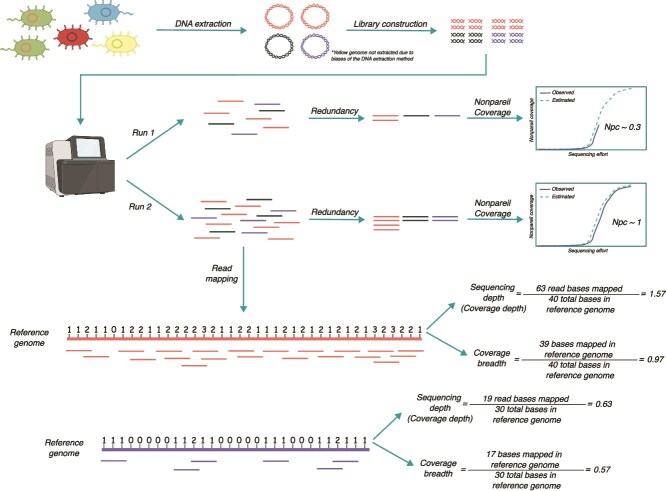
Schematic representation of the Npc calculation and its differences from sequencing depth (SD; or coverage depth) and sequencing breadth (or coverage breadth). All three metrics are commonly referred to as “coverage” in the literature, causing confusion about their exact meaning or differences. As depicted in the figure, Nonpareil is a database-independent approach as opposed to SD and coverage breadth that measure the frequency and extent of the reads mapped to a reference sequence, respectively. Note that Npc is calculated based on the redundancy of sequenced reads, with higher Npc values as redundancy increases. DNA present in the sample but not made into the library for sequencing (represented by the yellow genome) will not be accounted for in the Npc calculation. Partially created in BioRender. Aldeguer, B. (2025) https://BioRender.com/qmrhjlb.

Rodriguez-R and Konstantinidis developed Nonpareil in 2014 to calculate the diversity coverage of a metagenome based on read redundancy [14] (Fig. 1). Nonpareil coverage (Npc) is calculated by measuring the fraction of redundant reads in subsets of different sizes generating a redundancy curve, which is then fitted to a log-gamma distribution to estimate the sequencing effort needed to reach a given level of metagenome coverage [14]. Thus, by measuring the metagenome diversity coverage, Npc can be considered a proxy of sample diversity coverage. Npc does not correlate linearly with sequencing effort but instead depends on the biological complexity of the community, including factors such as species evenness and richness [14]. As a result, Npc represents a biologically-informed metric, in contrast to sequencing effort, which is a purely technical metric. Npc is commonly referred in the metagenomic literature as “coverage”, which can generate some confusion with other metrics that employ the same term, such as sequencing depth (SD) and breadth, also referred to as coverage depth and breadth, respectively. However, these are three distinct metrics that should not be conflated. Npc reflects the proportion of diversity captured in a metagenomic dataset, whereas SD and coverage breadth are metrics derived from analysis of read mapping against a reference sequence (Fig. 1). SD quantifies the frequency of reads mapped on the reference (e.g. 5X depth, or five sequencing reads per position on average), while coverage breadth measures the extent to which a reference sequence is covered by mapped reads (e.g. 50% breadth, or half of the length of the reference covered by reads; Fig. 1). To prevent confusion, we do not use the term “coverage” alone, but in conjunction with the appropriate type; i.e. diversity coverage, coverage breadth, and coverage depth. Additionally, to clarify potentially confusing concepts used throughout this manuscript, we define their meanings in Table 1.

**Table 1:** Definitions of potentially confusing terms employed in this manuscript.

Term	Definition
Diversity coverage	Fraction of the total diversity recovered in a sample, typically expressed as the fraction of cells belonging to species detected in the sample [range: 0–1]
Metagenome coverage	Fraction of the total DNA diversity recovered in a metagenome (proxy for diversity coverage) [range: 0–1]
Sequencing depth (or coverage depth)	Average number of times a given reference sequence is mapped by metagenomic reads [range: 0 – Inf]
Sequencing breadth (or coverage breadth)	Fraction of the reference sequence mapped by metagenomic reads [range: 0–1]
Rarefying	Process of subsampling a dataset once to obtain a single measurement
Rarefaction	Process of subsampling a dataset multiples time to obtain a central measurement
Normalization	Process of transforming a set of values relative to a given factor to ensure consistency, comparability and/or scale adjustment (e.g. divide sequencing depth by genome equivalents to calculate relative abundance).
Standardization	Process of estimating a given metric using consistent methods under uniform conditions to ensure comparability across datasets (e.g. estimate relative abundance at the same Npc).

The Nonpareil algorithm was significantly improved in 2018. The advancements included the use of k-mers to reduce the runtime of large metagenomes, the implementation of a sequencing error correction, and the introduction of the Nonpareil diversity metric [15]. A preliminary study was also conducted to explore the impact of Npc on gene detection and differential abundance analyses between metagenomes and concluded that two metagenomes should not be compared if they show more than 2-fold difference in metagenome coverage, otherwise spurious results could emerge [16]. However, the study included only two real, incomplete metagenomes with Npc of 0.64 and 0.75; a more comprehensive evaluation under different scenarios of Npc differences among datasets remained elusive. Further, the absence of an available ground-truth reference (i.e. the real abundances of features in the sequenced library to be known prior to the analysis) and the limited number of samples used significantly constrained the generalizability of the findings. Accordingly, while diversity coverage has been proposed as a standardizing strategy for ecological studies [10], exploring the potential of Npc to standardize metagenomic datasets warrants further investigation.

Standardization approaches of metagenomes have been largely limited to correct for uneven SD, either using methods borrowed from transcriptomics or rarefying [17]. However, the transcriptomics methods typically have assumptions that are violated by common metagenomes (see Supplementary Note 1). Rarefying, despite being likely the most common approach, showed a low performance for identifying differential abundance genes between metagenomes [17]. Therefore, more robust standardization methods specifically designed for metagenomics are needed to accurately normalize relative abundance values.

Here, we systematically evaluated the impact of uneven diversity coverage on metagenomic comparisons using both *in silico* and real metagenomes from natural environments. We further highlight the importance of metagenome coverage by showing that overlooking coverage differences has led several published studies to inaccurate conclusions about what features (e.g. genes, pathways, or taxa) differ in abundance and/or diversity (e.g. number of species) between microbiomes. Note that the recovery of microdiversity (e.g. the number of single nucleotide polymorphisms [SNPs] within a species) as a function of the sequencing effort (depth) applied is a distinct issue (than the macrodiversity we focus herein), and is dealt with elsewhere [18]. While previous Nonpareil studies primarily focused on software development, here we expand its use cases by introducing Npc coverage standardization as an approach to mitigate the effects of differences in diversity coverage between datasets on comparing feature abundances or diversities. We provide a straightforward decision tree to guide researchers in conducting robust feature abundance comparisons between metagenomic datasets, and explain why standardizing to the same sequencing effort (number of reads) is frequently not as robust as Npc standardization.

## Materials and methods

### Construction of in silico metagenomes

*In silico* (synthetic) metagenomes with known composition (ground truth) were simulated controlling for different biological characteristics, including evenness (even vs uneven), species richness (100 vs 1000 species) and microdiversity (1 vs 10 genome per species). Genomes were downloaded from NCBI and GTDB databases (Table S1). Short read sequences (150 bp) were simulated from genomes using Mason v2.0.9 [19] (−fragment-mean-size 150 −seq-technology illumina), which is known to accurately replicate the features of modern Illumina sequencers [20]. The number of reads per genome was calculated and the reads were subsequently subsampled appropriately to ensure that the resulting taxon rank curves conformed to a log-normal distribution, reflecting the patterns typically observed in real metagenomes [21]. All reads subsampled for each genome were then merged into a single file representing the *in silico* metagenome. The evenness of the distribution was determined by the location parameter (μ) of the log-normal equation (two and five for uneven and even distributions, respectively) and the ratio between the maximum and minimum value. Ratios of 10,000 and 500 were applied to get uneven and even distributions, respectively, based on the values we previously observed in real metagenomes. Note that for low abundance genomes the number of reads may be so low that only a fraction of the genome is represented in the final metagenome, similarly to real metagenomes. In order to facilitate reproducibility, the pipeline described here was implemented in the script “MetaG_simulator.py”, which is publicly available at https://github.com/baldeguer-riquelme/Nonpareil-coverage-standardization.

### Genome detection and relative abundance estimation

Detection of genomes/metagenome-assembled genomes (MAGs)/genes was based on read mapping analysis (i.e. assembly-independent). Specifically, relative abundance was calculated as SD divided by Genome Equivalents (GEQ), which normalizes for differences in the sequencing effort applied, length of MAGs/targets, and average genome size between the metagenomes [22] as well as for spurious matches [23, 24]. For this purpose, SD and GEQ were estimated by CoverM v0.6.1 (genome -p bwa-mem --min-read-percent-identity 95 --min-read-aligned-percent 70 --min-covered-fraction 10 --exclude-supplementary -m mean) [25] and MicrobeCensus v1.1.0 (default settings) [22], respectively. CoverM was also used to calculate mapped read counts which were converted to relative abundance as Read Per Kilobase per Million reads (RPKM). For simplicity, we used “relative abundance” to refer to the SD/GEQ metric, unless otherwise noted; e.g. for RPKM data. Plots were drawn in R using the ggplot2 library [26].

### Nonpareil coverage standardization

Metagenomes were subsampled to Npc values ranging from 0.1 to 0.9 in 0.1 increments. For this, the number of reads needed to reach a given Npc was calculated with the predict.Nonpareil.Curve function from the Nonpareil R library v3.5.3 using the npo files from Nonpareil v3.4.1 [15]. Then, reads were randomly subsampled to the target number of reads using reformat.sh. The approach was implemented in the Npc_standardization_manual.R script. Users can use this script to get randomly subsampled metagenomes with a given Npc and subsequently perform read mapping to features, etc. Alternatively, to simplify and speed up Npc standardization and reduce estimation error, relative abundance or count values can also be estimated using the Npc_standardization.R script. The .npo files from the Nonpareil analysis, the original relative abundance, GEQ or count values and the MAG/gene length should be provided to run the script. The proposed standardization approach consists of the following steps:

1. Estimation of the fraction of reads required to achieve a given Npc for each metagenome. For this purpose, the predict.Nonpareil.Curve function is used to calculate the number of reads at the target Npc, which is then divided by the total number of reads in the original metagenome.

2. The fraction obtained is multiplied by SD, GEQ or reads counts in the original metagenome to get the estimated relative abundance values in the subsampled metagenomes.

3. Finally, only MAGs/genes with a minimum 0.1X SD, which equates to ~10% coverage breadth according to the Lander–Waterman equation [27], are considered as present and used for comparisons between metagenomes. The remaining MAGs/genes are considered as undetected (i.e. zeros are assigned to MAGs/genes with SD <0.1X).

### Richness and differential abundance tests

Richness was assessed with three metrics, namely number of detected species (i.e. SD/GEQ > 0), Shannon index and Simpson index. Both Shannon and Simpson indexes were obtained with the diversity function of the vegan R package v2.6.8 [28]. Regarding differential abundance tests, the aggregated relative abundance of genomes belonging to the same phylum was compared between two metagenome types using the t.test() function from the stats R package. The significance of the statistical test as well as the direction (i.e. higher abundance in metagenome A than metagenome B or vice versa) in the subsampled metagenomes was compared to that obtained with the original metagenomes. When both results matched, the differential abundance test was labeled as accurate.

### Maximum acceptable difference in Npc (ΔNpc_max_)

To calculate the ΔNpc_max_ in a systematic fashion, we used the t.test function from stats R package v4.2.0 to compare the relative abundance at 0.7 Npc (reference) of each taxon at the phylum, class, order, and family level against the relative abundance of the same taxon in subsampled metagenomes showing stepwise decreasing Npc values (0.01 Npc per step). We ran this loop until the t.test became statistically significant. The difference between 0.7 and the specific Npc of the subsample considered was termed ΔNpc_max_, which was divided by the reference Npc (0.7) to calculate the maximum percentage of difference in Npc for statistically significant differences in feature abundance. For features with only one member, ΔNpc_max_ was calculated based on the minimum Npc at which that member was detected. This analysis was performed using the *in silico* metagenomes as well as seawater, human gut, freshwater, and peat soil metagenomes [24, 29, 30]. The approach was implemented in the Npc_max.R script, which can be used to calculate the ΔNpc_max_ of custom user data.

### Metagenome quality filtering, assembly, and binning

A total of 85 marine metagenomes representing surface to 200 meters deep previously published by Hawley and colleagues [31] were downloaded from NCBI using the prefetch and fastq-dump (−-minReadLen 50) tools of the SRA toolkit. Raw reads were quality-filtered using bbduk.sh v38.18 (qtrim = w,3 trimq = 17 minlength = 70 tbo tossjunk = t cardinalityout = t). Reformat.sh v38.18 separated pair-end reads in two files and Nonpareil v3.4.1 [14] was employed to calculate metagenome coverage and diversity on cleaned forward reads (-T kmer -f fastq -X 50000 -t 8). Cleaned reads were assembled with SPAdes v3.15.5 (--meta --only-assembler -t 24 -k 21,33,55,77,99 127) [32] and contigs longer than 1 kb were selected for binning with MaxBin2 v2.2.7 [33] and metaBAT2 v2.15 [34]. Note that we assembled the original but not the subsampled metagenomes. Metagenome-assembled genomes (MAGs) were dereplicated with dRep v3.4.3 (−sa 0.95) [35] yielding a total of 219 species-like MAGs that were quality-assessed using CheckM v1.2.2 [36] and taxonomically classified with GTDB-tk v2.3.2 (r214) [37].

### Antibiotic resistance genes relative abundance in wastewater metagenomes

To showcase the impact of Npc standardization in avoiding biased biological results due to differences in metagenome coverage, the dataset analyzed in Zhang *et al.*, 2021 [38] was reanalyzed here following the same pipeline they used but introducing standardization. Briefly, raw reads were downloaded with prefetch and fastq-dump and cleaned with fastp v0.21.0 (default parameters). Npc was calculated, and metagenomes subsampled to the same Npc. The abundance of antibiotic resistance genes (ARGs) in the original as well as the subsampled metagenomes was calculated with ARGs-OAP v3.2.4 [39] (default parameters) as in Zhang and colleagues. The estimateR function from the vegan R package [28] was employed to calculate Chao1 index using as input the unnormalized counts for ARG subtypes provided by ARGs-OAP. Differential abundance test was performed with the t_test function of the R package rstatix [40] using the ARG subtypes abundance normalized by cell counts. Plots were drawn on R with ggplot2 v3.4.2 [26].

## Results and discussion

### Relative abundance and richness estimates can be affected by uneven diversity coverage

The motivation for this manuscript emerged from our own experience and mistakes. While analyzing metagenomes from complex peatland soils, we detected higher potential for oxygen respiration in the anoxic zone below the water table than in the surface oxic zone, which was apparently unexpected. Further investigation revealed differences in Npc between metagenomes as the source of this bias. This finding further highlighted the importance of addressing diversity coverage in metagenomic studies, motivating us to write this manuscript to raise awareness within the scientific community.

We first showcase the impact of differences in sequencing effort and Npc on derived conclusions by comparing the relative abundances of genomes in *in silico* metagenomes displaying different complexities in terms of evenness (high vs low evenness; we refer to these as even vs uneven metagenomes below, respectively), species richness (100 vs 1,000 distinct species), and microdiversity (1 vs 10 distinct genomes per species). The abundances of features in these metagenomes are thus known. The even and uneven datasets were produced using log-normal models of species abundances with different values of the location parameter (μ) and maximum/minimum abundance ratios (i.e. μ = 2 and max/min ratio = 10,000 vs μ = 5 and max/min ratio = 500, respectively). Note that high evenness does not mean equal abundances between species, which is rarely—if ever—observed in natural communities, but rather more even abundances compared to the uneven metagenomes. For assessing the impact of microdiversity, 10 genomes showing between 95% and 99.5% ANI among themselves were used. Nonpareil curves revealed evenness and species richness influence Npc estimation while microdiversity had a minimal impact (Fig. S1), presumably because the default parameters of Nonpareil are calibrated to assess redundancy at the 95% nucleotide identity (i.e. the species level). If intra-population (intraspecies) diversity is larger than this level, then such microdiversity would have a significant impact on Npc, and/or the default of 95% nucleotide identity of Nonpareil has to be adjusted accordingly. We did not explore this further as natural populations with greater microdiversity are rather uncommon [41]. Consequently, our subsequent analyses were limited to metagenomes exhibiting distinct levels of evenness and species richness.

**Figure 2:**
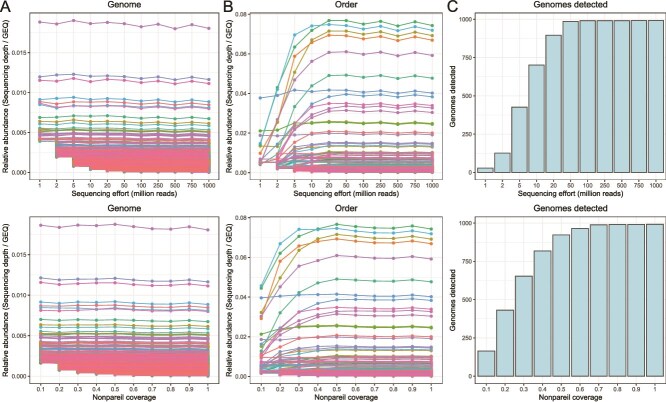
Impact of sequencing effort (top) and Npc (bottom) on the relative abundance estimate of individual genomes (A), groups of genomes (B), and the total number of detected genomes (C). Data were derived from an *in silico* metagenome with 1,000 species and an uneven genome distribution (Uneven1000sp). Each line in (A) and (B) represents the relative abundance of an individual genome or the aggregated relative abundance of a group of genomes within the same order, respectively. Note that the relative abundances of individual genomes remain stable (A), while the relative abundances of orders increase with higher sequencing effort and Npc (B), driven by the detection of more of their member genomes (C). The same picture was obtained from metagenomes with different richness and evenness.

The genome relative abundances were robustly estimated using read recruitments as SD normalized by GEQ (SD/GEQ, see Methods) in the complete metagenomes as well as in the subsampled metagenomes of varying Npc levels and number of sequences (Fig. 2A). Note that the reads were mapped back to the genomes used to simulate the metagenomes with no assembly step involved. The taxon rank curves of each metagenome type and replicate are shown in Fig. S2. The relative abundances of MAGs belonging to the same taxon (e.g. same order) were added up to represent the abundance of the taxon in each sample and the derived taxon abundances were directly compared between the complete metagenome and subsampled metagenomes at varying Npc levels. We consistently observed, across multiple taxa, increasing differences in their relative abundance in subsampled metagenomes of decreasing Npc compared to the original metagenome (Fig. 2B). For instance, the aggregated relative abundance of MAGs belonging to the order *Flavobacteriales* decreased by ~74% in metagenomes of Npc = 0.9 vs. 0.1. These results were initially counterintuitive because metagenomes, and their subsamples, are thought to be random subsets of the sequenced DNA; hence, relative abundance should be similar, if not identical, in subsamples. Further analysis revealed that many genomes were not detectable in subsampled metagenomes of low Npc coverage due to their relatively low abundance (Fig. 2C). Consequently, in the example of the *Flavobacteriales*, the drop of 74% in abundance in the completely sequenced metagenome vs a metagenome that was sequenced at Npc = 0.1 was simply due to diversity coverage differences, not actual differences in abundances. Collectively, these results indicated that sensitivity in detecting members of the feature, in this case a group of MAGs belonging to the same order, rather than the abundance of individual MAGs that make up the order, was responsible for the differences in the aggregated relative abundance of the feature observed as a function of the Npc and sequencing effort (Fig. 2B). In other words, the relative abundance of the individual MAGs remains stable in subsamples, as anticipated, but the number of detected MAGs increases with sequencing effort (or Npc coverage), which translates to higher aggregated abundance of the corresponding feature that the MAGs are assigned to.

### Npc standardization mitigates errors associated with uneven metagenome coverage

**Figure 3:**
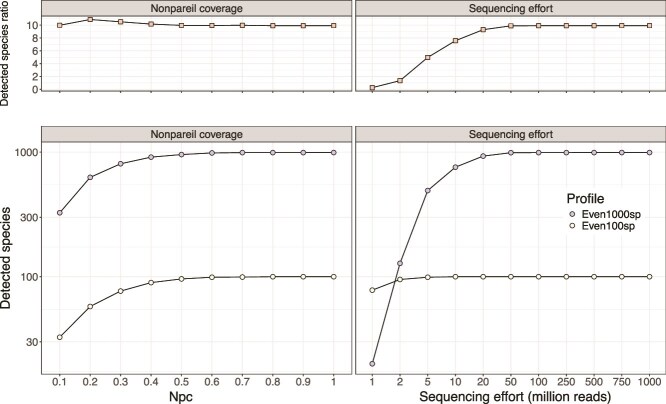
Number of detected species in metagenomes standardized to the same Npc (left) and sequencing effort (right). The real species ratio is 10 and the observed species ratio is shown on the top panels. Note that the Npc standardized metagenomes accurately capture the real richness differences between the metagenomes, contrasting with the sequencing effort standardization, which even provides the opposite result to the ground truth at relatively low sequence effort (1 million reads), showing higher diversity for the Even100sp than the Even1000sp datasets.

The number of MAGs (or individual genes) detected can vary with metagenome coverage (Fig. 2C), which can significantly impact diversity and differential abundance analyses, leading to spurious or unreliable results. This bias can be minimized by standardizing to the same Npc, and this approach is advantageous compared to the common alternative of sequencing effort standardization, as we show below. Specifically, Even1000sp metagenomes contain 10 times more species than Even100sp metagenomes, a richness ratio accurately captured when standardizing to the same Npc but not when Npc was unequal, or when metagenomes were standardized to the same sequencing effort (Fig. 3). Furthermore, Npc standardization consistently produced the correct qualitative result, showing higher diversity for Even1000sp compared to Even100sp across sub-samplings. In contrast, sequencing effort standardization reversed this result at relatively low sequencing efforts, incorrectly showing higher diversity for Even100sp at 1 million reads, underscoring the limitations of read-based standardization. This strikingly misleading picture of the sequence effort standardization approach was due to the fact that the most abundant genomes in the Even100sp dataset were more abundant (and thus detectable) than the most abundant genomes in the Even1000sp dataset (see taxon rank curves in Fig. S2). We observed similar results with the same datasets when we performed alpha-diversity comparison using the Shannon and Simpson indexes (Fig. S3 and S4), further underscoring the robustness of Npc standardization. Comparable results were obtained based on the analyses of the Uneven1000sp and Uneven100sp datasets, although richness differences between the two datasets became inaccurate at low Npc values (<0.4) (Fig. S5). These findings highlight the strong qualitative (which dataset is higher/lower) and quantitative (how much higher/lower) benefits of standardizing to the same Npc for diversity comparisons.

**Figure 4:**
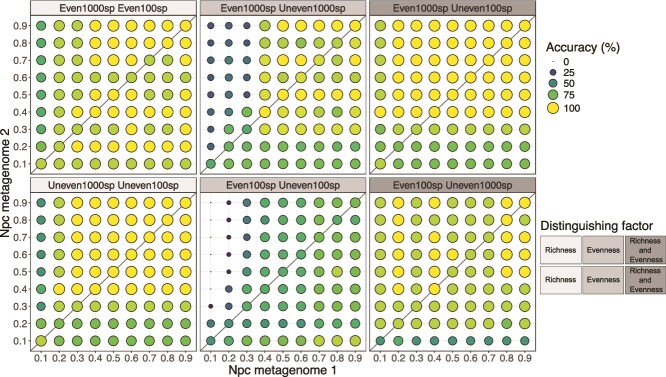
Accuracy of differential abundance tests across subsampled metagenomes of varying Npc. Aggregated relative abundances at the phylum level in the subsampled datasets were statistically compared between metagenome types. The significance of the test was compared to that obtained with the original metagenomes in order to assess the accuracy of the result (e.g. *Acidobacteriota* abundance higher in metagenome 1 than in metagenome 2). A total of nine phyla were compared, with the exception of Even1000sp vs. Uneven1000sp with 17 phyla. Each panel displays the result for one metagenome type pair (see panel titles). The diagonal line shows the comparison between metagenomes standardized to the same Npc. Abundances were compared based on a pairwise t-test among metagenome types with *P*-value adjusted using the Benjamini–Hochberg (BH) correction. The dot size and color indicate the percentage of tests performed that correctly detected the difference in abundance based on the complete metagenomes (i.e. accuracy; see figure key). Note that accuracy increases as Npc increases.

To further quantify the importance of Npc standardization, we used the aggregated relative abundance at the phylum level to perform differential abundance analyses on metagenomes subsampled to a range of Npc values and using the results of the original (not subsampled) metagenomes as reference (i.e. correct result). As expected, the fraction of accurate tests increased with increasing Npc for all six metagenome combinations (even vs. uneven, 100sp vs. 1000sp, and their combinations; Fig. 4). This result was further reinforced by Bray-Curtis distances, which showed increasing divergence in the phyla abundance results between subsampled metagenomes and the original (full) metagenomes as Npc decreased (Fig. S6). In addition, differential abundance tests with Npc standardized metagenomes showed higher accuracy compared to unequal Npc metagenomes. For example, the comparison between even and uneven metagenomes with 1000 species yielded an average accuracy of 73.7% for unequal Npc metagenomes that increased to 86.3% when Npc standardized metagenomes were used, representing a ~13% improvement in accuracy (Table 2). However, while Npc standardization minimizes inaccuracies, it does not eliminate them entirely, as the accuracy was in all cases below 100%, reinforcing the importance of reporting Npc to contextualize results.

**Table 2:** Accuracy of differential abundance tests using metagenomes standardized to the same (equal Npc) or different Npc (unequal Npc). The underlying data is the same displayed in Fig. 4.

Metagenomes A			Metagenomes B			Average accuracy (%)		Improvement (equal Npc—unequal Npc; %)
# species	Evenness		# species	Evenness	Distinguishing factor	Unequal	Equal Npc	
1000	Even		1000	Uneven	Evenness	73.7	86.3	12.6
100	Even		100	Uneven		57.3	66.7	9.4
1000	Uneven		100	Uneven	Richness	89.0	95.1	6.0
1000	Even		100	Even		91.5	93.8	2.3
1000	Even		100	Uneven	Evenness and richness	92.9	95.1	2.2
100	Even		1000	Uneven		88.0	88.9	0.9

### Npc standardization improves aggregated abundance estimates in metagenomes from natural environments

**Figure 5:**
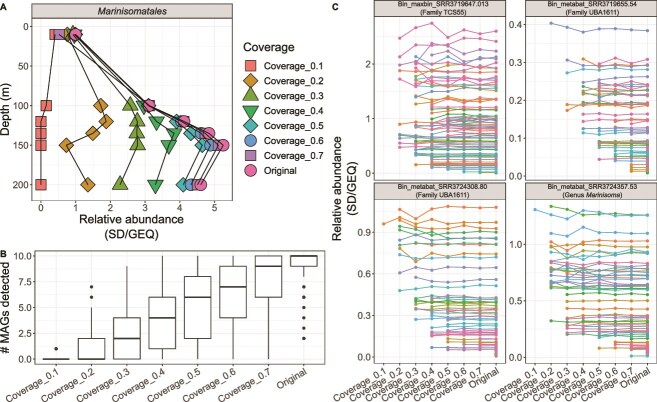
Effect of metagenome coverage on the aggregated relative abundance of a feature. (A) Aggregated relative abundance (*x*-axis), calculated as SD divided by GEQ, of a group of MAGs belonging to the order *Marinisomatales* is shown (*x*-axis) against the depth of the water column that the corresponding metagenomes were obtained from (*y*-axis). The average abundances from replicate metagenomes at each depth (10 m *n* = 8 replicates; 100 m *n* = 15; 120 m *n* = 12; 135 m *n* = 11; 150 m *n* = 15; 200 m *n* = 16) and subsampled metagenomes are shown. Note the increase in relative abundance as metagenome coverage increases. (B) Number of *Marinisomatales* MAGs (*y*-axis) detected in subsampled metagenomes (*x*-axis). Note the increase in the number of MAGs detected as metagenome coverage increases. (C) Relative abundance (*y*-axis) of individual MAGs belonging to *Marinisomatales* across subsampled metagenomes (*x*-axis). Each panel represents an individual MAG and each line and color indicate the relative abundance of such MAG across sub-samplings of the same sample. Note the consistency of relative abundance values across subsampled metagenomes but also that several MAGs become undetectable at low coverage subsamples (and thus, after this point, they do not contribute to the relative abundance of the *Marinisomatales* order).

To demonstrate that the impact of Npc on the aggregated relative abundance of a group of MAGs (e.g. an order) is not due to the design (e.g. species abundance curve) of our *in silico* metagenomes, we also examined metagenomes from natural environments. Specifically, we used the metagenomes from the ocean depth profile produced by Hawley and colleagues [31]. The aggregated relative abundance of a group of MAGs belonging to the order *Marinisomatales* was estimated based on the SD/GEQ metric in the complete metagenomes and their subsampled datasets (Fig. 5). A set of subsampled metagenomes standardized to the same Npc produced a peak in *Marinisomatales* abundance at 150 meters, in agreement with the full, high-coverage metagenomes (average Npc = 0.8; Fig. 5A), while the profile based on unequal Npc revealed the opposite trend as an effect of metagenome coverage, not actual biological/ecological differences (Fig. S7). These results were attributable to the increasing detection of MAGs with higher Npc (Fig. 5B) rather than to changes in the relative abundance of individual MAGs. In fact, the relative abundance of MAGs remained stable across subsampled datasets as expected, given the random nature of metagenomes and our approach to create subsampled datasets, until MAGs became nondetectable in low coverage metagenomes (Fig. 5C). We also observed that subsampled metagenomes with equal coverage at medium-to-high Npc (≥0.5) showed the same depth-profile trends (i.e. an abundance peak at 150 m) compared to the full metagenomes (average Npc = 0.8) while those at 0.4 Npc or below displayed divergent trends (i.e., abundance peak at depths other than 150 m; Fig. 5). This observation is in line with the 0.6 Npc threshold previously proposed to ensure adequate metagenome coverage and assembly of the sampled community and thus, meaningful biological comparisons between metagenomes [16].

### Published microbiome studies often report unreliable results

To address the impact of unequal Npc in published microbiome studies, we reanalyzed previously published richness and abundance results. For example, for features that involve genes, Zhang *et al.*, 2021 [38] apparently overestimated the ARG diversity found in effluent water samples of wastewater treatment plants (WWTP) due to the higher Npc of these samples compared to influent and sludge samples (Fig. S8A, B). Consistently, half of the ARGs subtypes identified as differentially abundant between different WWTPs previously [38] displayed a significantly different pattern when the metagenomes were subsampled to the same Npc (Fig. S8A, C).

Such cases are probably widespread in recent microbiome literature, unfortunately. Based on a literature search, the number of manuscripts performing differential abundance analysis with metagenomic data has increased from 89 manuscripts in 2014 to 2,880 in 2023 (Fig. S9). However, only 11 manuscripts out of the total 2,880 (0.38%) in 2023 considered Nonpareil in their analyses. Therefore, despite previous Nonpareil manuscripts having been cited over 600 times at the time of this writing, the tool has not been employed in the context of differential abundance analyses. In addition, popular statistical tools for differential feature abundance analysis of amplicon or transcriptomic data, such as metagenomeSeq, DESeq2, and edgeR [17, 42–44], apply different approaches to normalize read counts based on library size (e.g. CSS, RLE, and TMM) but do not incorporate differences in gene/genome length nor diversity coverage and make assumptions that are violated by metagenomic data (see Supplementary Note 1), limiting their usefulness for such data.

### The need for Npc standardization depends upon the relative abundance of the target feature

We next opted to answer the question of how similar, in terms of Npc, the metagenomes should be in order to provide reliable relative abundance comparisons. For this, we calculated the maximum difference in Npc, as a fraction of the sample’s Npc, which provided statistically identical results in terms of the relative abundance of a feature between the original and the subsampled metagenomes (ΔNpc_max_; see Methods); i.e. the subsample provided unbiased results. The histogram of ΔNpc_max_ values showed a wide and even distribution with no predominant peaks (Fig. S10), suggesting that this parameter is highly variable, and there is no universal cutoff. Instead, we observed that the ΔNpc_max_ positively correlated with the average relative abundance of the feature of interest for the *in silico* metagenomes (R^2^ = 0.55–0.27, *P*-value<.001, Fig. S11) as well as for various metagenomes from different environments (R^2^ = 0.57, *P*-value<.001, Fig. S12), revealing no sample- or habitat-specific biases. These findings suggested that assessing abundant features, and thus robustly detected even in subsampled metagenomes, may not be biased when comparing metagenomes of different Npc while assessing low abundance features is more likely to be biased even when the ΔNpc is relatively low. For example, the estimate of abundance for a taxon with an abundance of 0.1% (measured as SD/GEQ*100) of the total metagenome will not be biased unless ΔNpc is higher than 50%, based on the marine metagenomes with medium-to-high Npc used here. In contrast, the estimate for a taxon with an abundance of 0.02% will be biased if the ΔNpc is higher than 10%. The correlations shown on Fig. S12 can be useful to identify situations where this bias will be important to take into account or not.

### Recommendations for comparative metagenomic analyses

**Figure 6:**
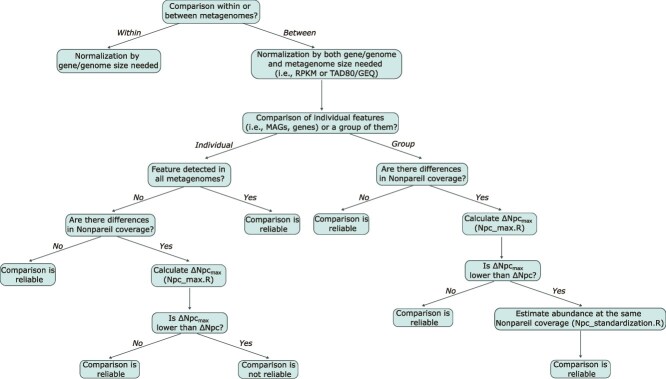
Decision tree to guide analysis of differential feature abundance or diversity between metagenomes.

Assessing features with single members (e.g. a MAG or a specific gene allele) is not problematic with respect to diversity coverage because metagenomes are random samples of diversity by nature. Hence, as long as the MAG/gene is confidently detected, relative abundance should be preserved in subsampled metagenomes. For features with multiple members (e.g. a bacterial genus or a gene family), sequencing high-coverage metagenomes (e.g. Npc > 0.9) is the most effective way to minimize metagenome coverage-associated biases. However, obtaining high metagenome coverage is not practical for several environments, such as soils, or studies with many samples. For such cases, data standardization is likely necessary for comparative purposes, but determining the optimal standardization approach in any given situation may be challenging. To assist researchers with this challenge, we provide a decision tree that can be used to guide comparative metagenomic analyses under different scenarios (Fig. 6). Specifically, caution is needed when the metagenome coverage is not high (e.g. Npc < 0.9), and there are differences between the Npc of the metagenomes being compared. In such cases, we recommend first calculating the ΔNpc_max_ of the feature of interest (a group of MAGs/genes representing a taxon/function) based on the metagenome(s) with the highest Npc in the comparison performed using the Npc_max.R script. If the ΔNpc_max_ is lower than the actual ΔNpc between the metagenomes compared, the comparison might be biased due to differences in metagenome coverage. In the latter case, we recommend calculating the relative abundance of the feature in metagenomes that have been standardized by subsampling to the same Npc (e.g. to the lowest Npc) (Fig. 6). Relative abundance and/or diversity values obtained after Npc standardization can then be used for more reliable comparative analyses. Metagenome subsampling and read mapping to calculate abundances in subsampled metagenome can be computationally expensive and requires experienced users. To assist with this step, we provide a fast, easy-to-use script (Npc_standardization.R) that accurately estimates relative abundance in subsampled metagenomes at a given, user-defined Npc using the abundance in the original complete metagenome. The abundance predictions of our tool showed perfect correlation (R^2^ = 1) with minimal error (~1%) when compared to the observed abundance values obtained by actually subsampling and mapping the metagenomes reads (Fig. S13), and thus can be used for direct comparisons between metagenomes. Note that the Npc standardization approach presented here is useful for richness and differential abundance comparisons (i.e. macrodiversity). However, when the focus is on comparing differences in microdiversity within a species or population, standardization to the same SD of the target species/population should be employed [18]. Additionally, highly incomplete (e.g. <50% completeness) or contaminated (e.g. >10% contamination) MAGs could provide unreliable relative abundance estimates, as discussed previously [45]. Therefore, we recommend using only medium to high-quality MAGs as defined by MIMAG standards [46], for the types of comparative analyses described above.

It should be noted that even after Npc standardization (for metagenomes of uneven metagenome coverage) the relative abundances of features obtained could differ somewhat from the actual abundances in the completely sequenced samples (Figs 2 and 5). Thus, Npc standardization can make comparative analyses more robust but cannot guarantee the perfect accuracy of the resulting estimates, especially if standardizing to a relatively low coverage (e.g. Npc < 0.3; see Figs 4, S3, S4, S5 and S6), and the level of its success depends on the abundance distribution of the members of the feature being assessed (which determines at what sequencing effort/depth the features are detectable or not). The abundance distribution is typically unknown; therefore, dealing, in full, with this limitation is currently not feasible or requires more data (e.g. Npc > 0.9). Further, despite some controversy regarding the potential loss of statistical power with data subsampling [4, 9, 47], avoiding false positives commonly outweighs the risk of missing significant comparisons (false negatives), making rarefaction a pragmatic and advantageous approach compared to the alternative of analyzing the data as is. Moreover, since the abundance predictions generated by the Npc_standardization.R script produce maximum-likelihood estimates based on the complete data, the expected central value for abundances is adjusted to be comparable across datasets without a corresponding increase in the estimation error and no data is discarded. Indeed, we observed ~2.1% average deviation within the observed SD values after 10 random subsampling of the same metagenome, which is in the same range or even higher than the ~1% average deviation of the same abundance value estimated by our script. Therefore, we recommend using this standardization approach and our method outlined above when possible, and resorting to random subsampling only for more complex cases not covered by our tool (e.g. when users want to assemble each subsample or create read recruitment plots).

## Conclusion

In summary, we suggest to: (i) aim to sequence high coverage metagenomes to minimize the fraction of the community not covered, (ii) be aware of and calculate metagenome coverage, (iii) standardize the data to the same Npc when there are substantial differences in Npc (e.g. ΔNpc_max_ < ΔNpc), and (iv) be aware that results from differential abundance or diversity analyses for the obtained Npc may differ in higher coverage metagenomes. Accordingly, we expect that the recommendations provided will help to minimize biases in comparative metagenomic studies, thereby facilitating the generation of quantitative, standardized, and meaningful results.

## Supplementary Material

Aldeguer_Riquelme_et_al_2025_Suppl_Material_ycaf140

Table_S1_ycaf140

## Data Availability

The scripts and instructions to run them are available at https://github.com/baldeguer-riquelme/Nonpareil-coverage-standardization.
