# Supplementary material for: Differences in metagenome coverage may confound abundance-based and diversity conclusions and how to deal with them
Source: ISME Commun. 2025 Sep 10;5(1):ycaf140. doi: 10.1093/ismeco/ycaf140 (PMC12477595; doi:10.1093/ismeco/ycaf140)
Supplement: Aldeguer_Riquelme_et_al_2025_Suppl_Material_ycaf140 [file aldeguer_riquelme_et_al_2025_suppl_material_ycaf140.pdf]

Differences in metagenome coverage may confound abundance-based and diversity conclusions and how to deal with them

Borja Aldeguer-Riquelme[1,2#], Luis M. Rodriguez-R[3#], Konstantinos T. Konstantinidis[1#]

[1]School of Civil & Environmental Engineering and School of Biological Sciences, Georgia Institute of Technology, Atlanta, GA 30332, USA

[2]Department of Biomedicine and Dentistry, European University of Andalucia, Málaga, Spain

[3]University of Innsbruck, Innsbruck, Austria.

#Correspondence should be addressed to BAR, LMR and KTK (briquelme3@gatech.edu, lmrodriguezr@gmail.com and kostas@ce.gatech.edu)

Supplementary Note 1. Current metagenome normalization strategies and why they are not appropriate.

Normalization approaches originally developed for transcriptomics, such as the Trimmed Mean of M-values (TMM) and Relative Log-Expression (RLE), have been adopted for metagenomics studies to correct for uneven sequencing depth [1]. These methods calculate scaling factors based on gene abundance (counts). On one hand, TMM normalizes gene abundance by comparing each sample against a reference sample. For each sample, the scaling factor is computed as the weighted trimmed mean value of the differences of the log-transformed gene abundances between the sample and the reference. On the other hand, RLE derives its scaling factor from a pseudo-reference value, calculated as the geometric mean of gene abundances across all samples included in the study. Although widely validated for transcriptomic data, these approaches rely on assumptions that do not hold for metagenomic data. First, they implicitly assume that the full set of reference genes/genomes is available. This is reasonable for human transcriptomics, where the complete genome is known, and nearly all transcripts can be mapped. However, in metagenomics, reference genes/genomes are generally incomplete or unknown and typically only a fraction of reads will map to the sequences selected as the reference. Given that TMM and RLE rely on the abundance of detected genes to calculate the scaling factors, only mapped reads are considered in the calculation whereas the fraction of unmapped reads is ignored. More importantly, such a fraction of unmapped reads could vary between metagenomes, which could potentially bias the results. Second, these approaches do not account for the fraction of diversity sequenced and assume the diversity coverage is high, and that few genes remain undetected. While this assumption holds for low-diversity samples (e.g., human tissue), it is often violated in metagenomics, where only a fraction of the total diversity is typically sequenced. Third, TMM requires a reference sample selected by the researcher to calculate the scaling factor. It is reasonable to define the reference sample as the "control" sample in a transcriptomic study, but it could be an arbitrary decision for metagenomics, potentially impacting results. Fourth, TMM assumes that most genes are not differentially abundant, which is a valid assumption for transcriptomic studies of a single genome. However, in metagenomic studies, the high complexity of microbial communities often leads to a greater proportion of differentially abundant genes, challenging this assumption [2]. Beyond scaling methods, rarefaction [1] consists on randomly subsampling the sequencing dataset without replacing until a uniform sequencing effort is achieved across metagenomes. Rarefying could be considered the most common standardization approach in metagenomics, as samples sequenced within the same study typically undergo a similar sequencing effort. In other words, researchers generally aim to sequence the same number of reads across all samples within a study, unintentionally but effectively rarefying the metagenomes. Despite being common, rarefying showed a low performance for identifying differential abundance genes between metagenomes [1].

<u>References</u>

1. Pereira MB, Wallroth M, Jonsson V, Kristiansson E. Comparison of normalization methods for the analysis of metagenomic gene abundance data. *BMC Genomics* 2018; 19: 274.
2. Weiss S, Xu ZZ, Peddada S, Amir A, Bittinger K, Gonzalez A, et al. Normalization and microbial differential abundance strategies depend upon data characteristics. *Microbiome* 2017; 5: 27.

Supplementary Figures

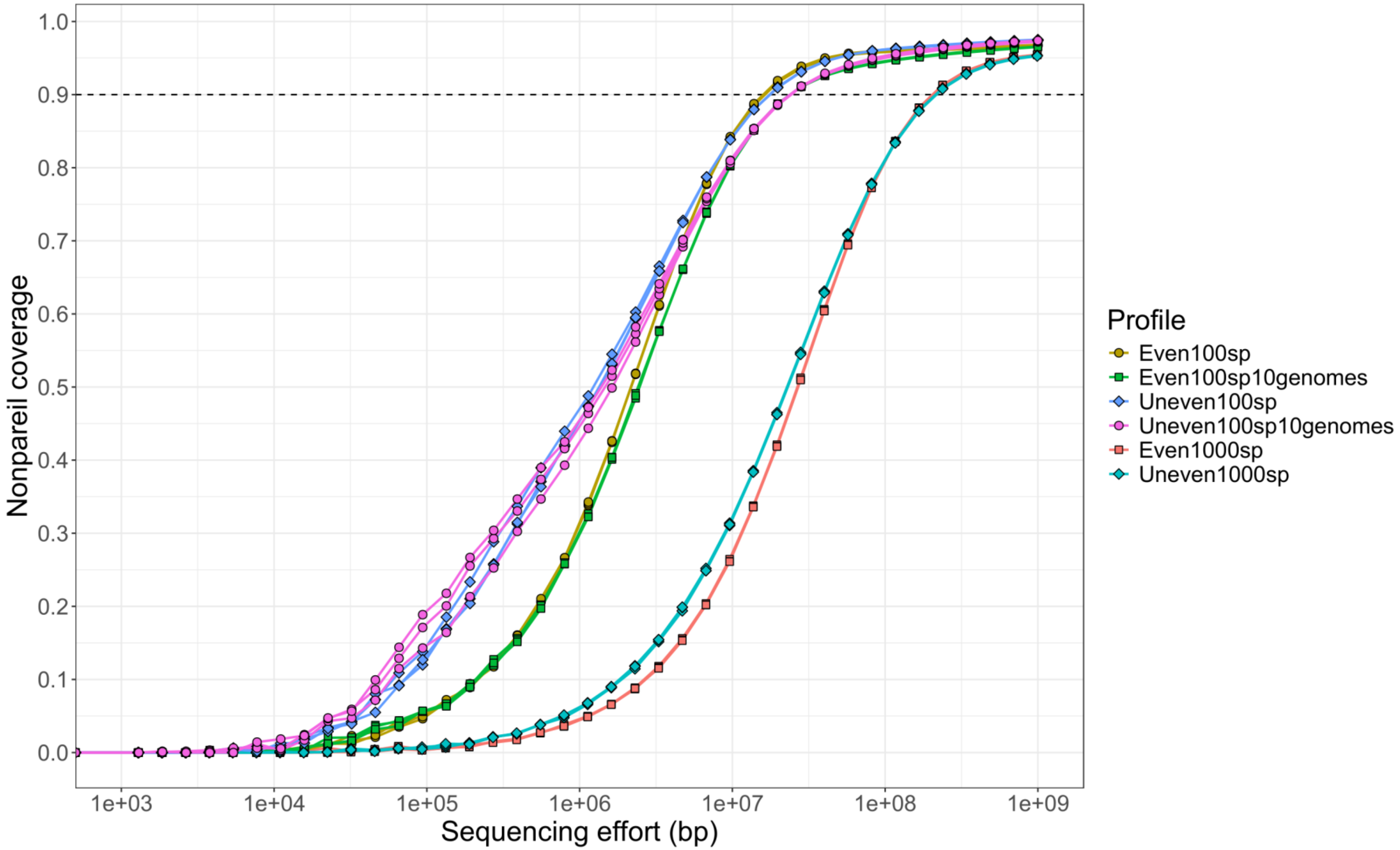

Figure S1. Nonpareil coverage (y-axis) as a function of sequencing effort of the *in silico* metagenomes produced in this study. The metagenomes showed differences in terms of evenness (high vs low evenness, that we refer to as even and uneven, respectively), species richness and microdiversity (see figure key).

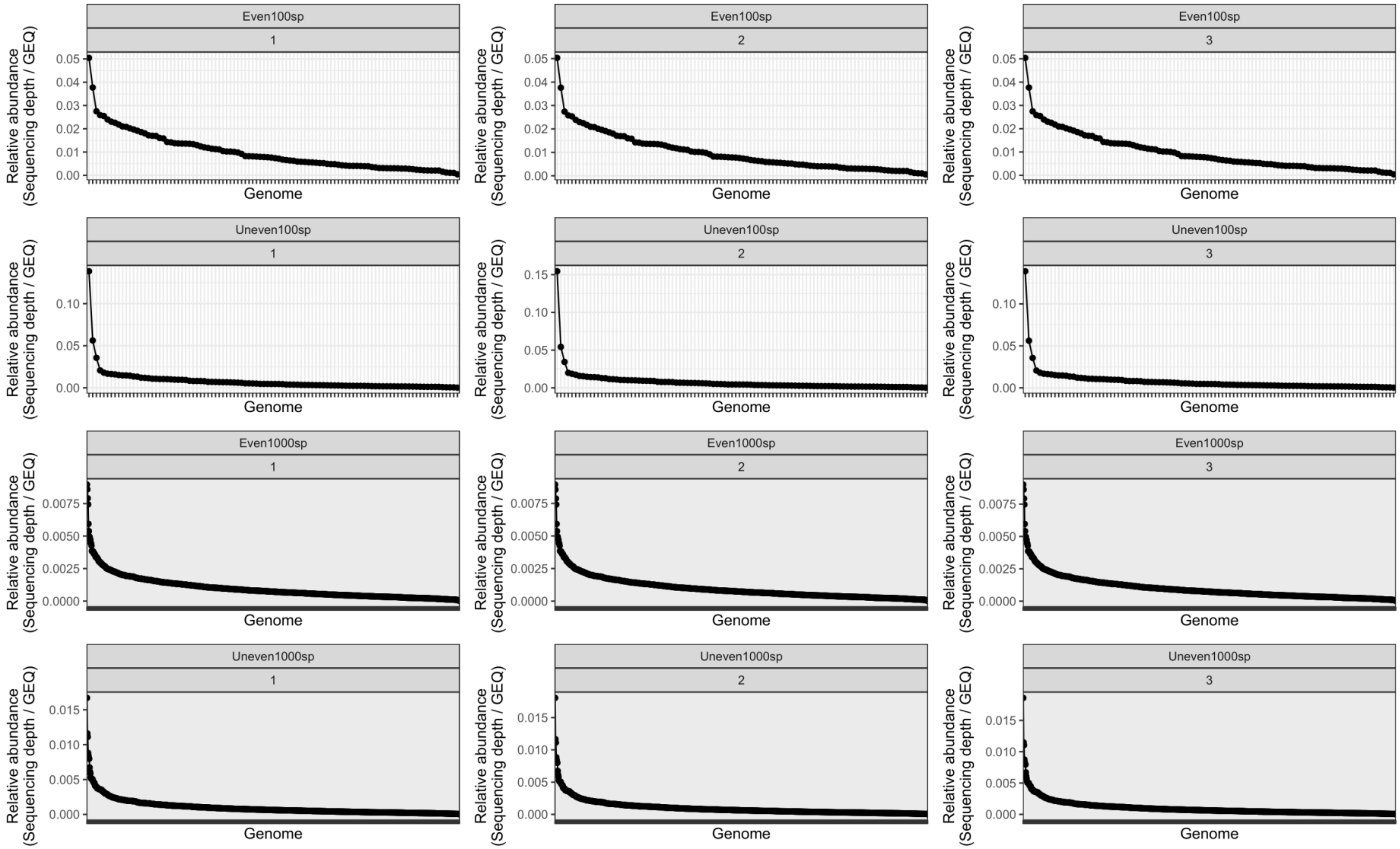

Figure S2. Taxon rank abundance curves for each *in silico* metagenome type (top panel title) and replicate (bottom panel title) produced in this study. Note that genome abundances are more equal between them for the "Even" than for the "Uneven" metagenomes.

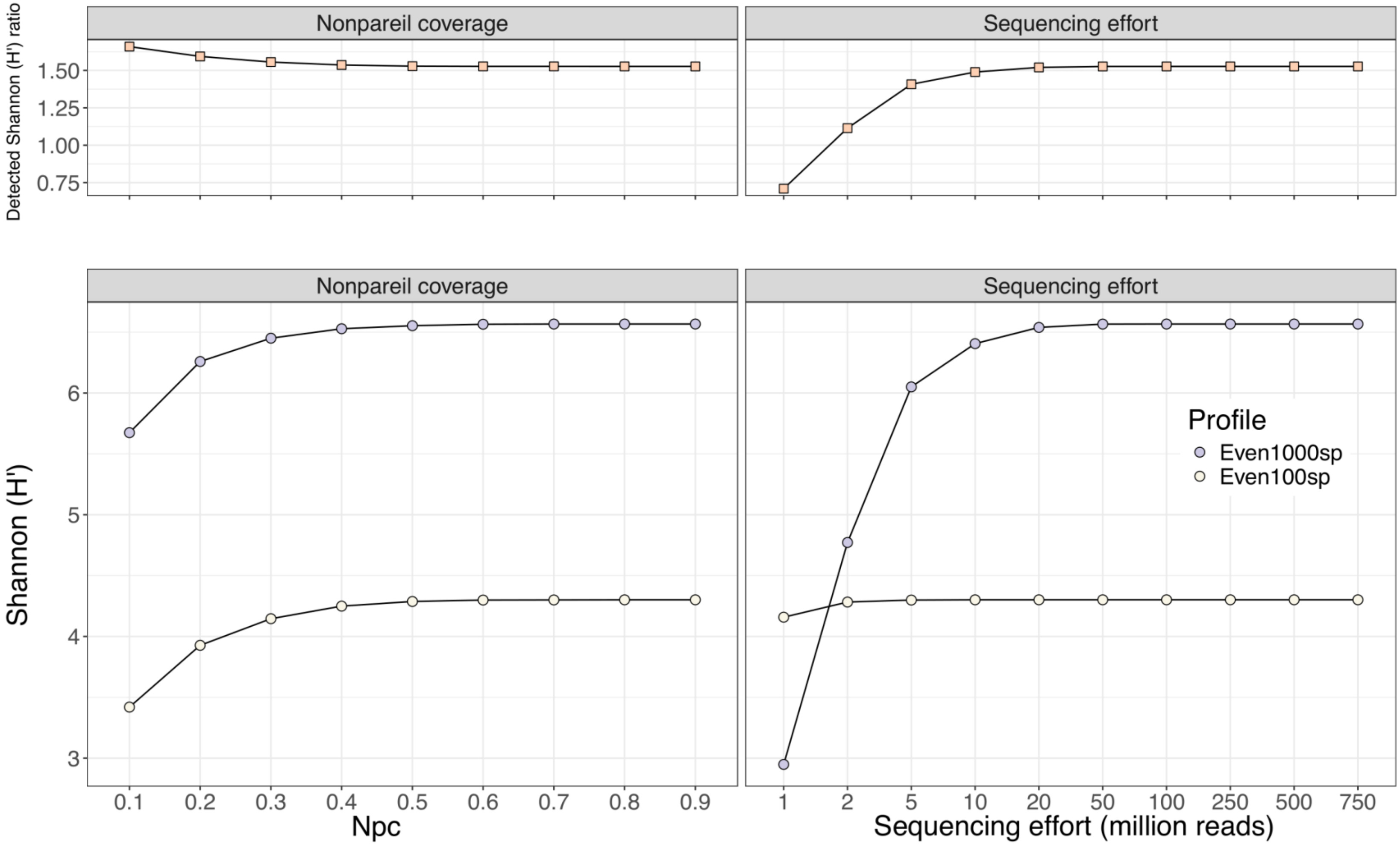

Figure S3. Shannon index in metagenomes standardized to the same Nonpareil coverage (left) and sequencing effort (right). The detected Shannon ratio is shown on the top panels. Note that Nonpareil coverage standardized metagenomes accurately capture the qualitative (which dataset is higher/lower) and quantitative (how much higher/lower) richness differences between metagenomes. Note that the estimated Shannon ratio is not highly accurate at very low Nonpareil coverage. On the contrary, sequencing effort standardization even inverts the qualitative differences in Shannon index showing higher diversity of Uneven100sp respect to Uneven1000sp when standardized to 1 million reads and generally shows much less accuracy.

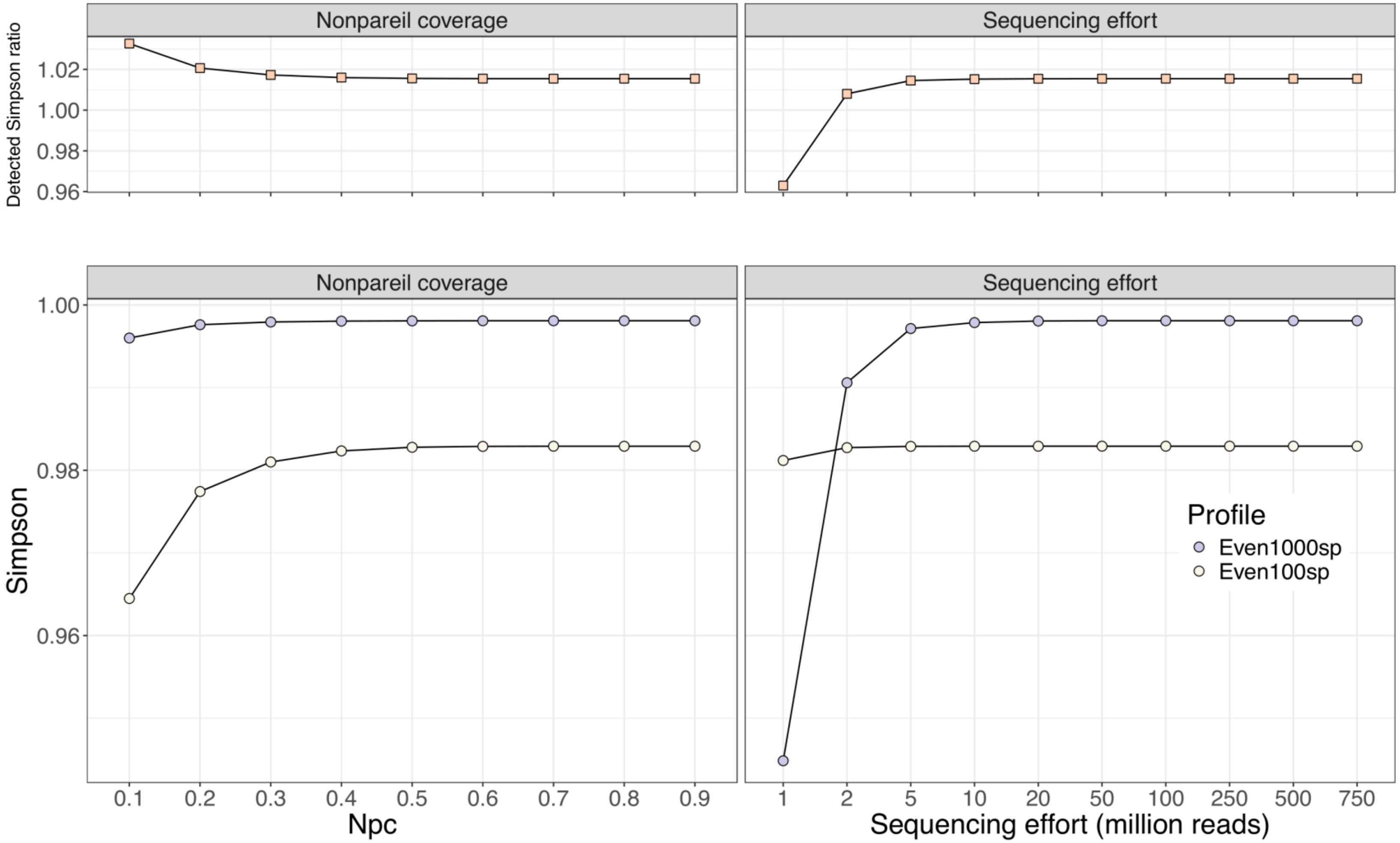

Figure S4. Simpson index in metagenomes standardized to the same Nonpareil coverage (left) and sequencing effort (right). The detected Simpson ratio is shown on the top panels. Note that Nonpareil coverage standardized metagenomes accurately capture the qualitative (which dataset is higher/lower) and quantitative (how much higher/lower) richness differences between metagenomes. Note that the estimated Simpson ratio is not highly accurate at very low Nonpareil coverage. On the contrary, sequencing effort standardization even inverts the qualitative differences in Sampson index showing higher diversity of Uneven100sp respect to Uneven1000sp when standardized to 1 million reads and generally shows much less accuracy.

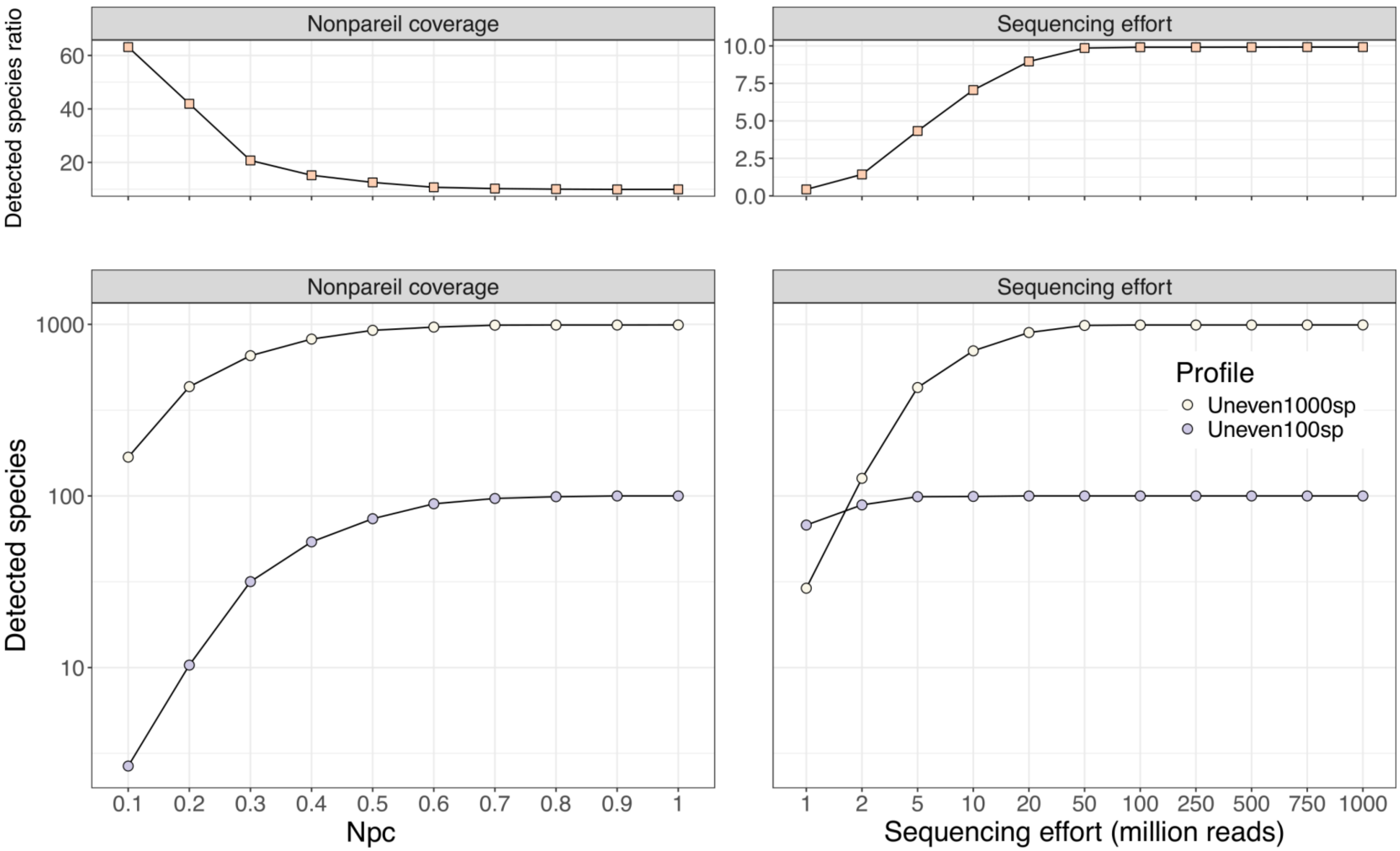

Figure S5. Number of detected species in metagenomes standardized to the same Nonpareil coverage (left) and sequencing effort (right). The detected species ratio is shown on the top panels. Note that Nonpareil coverage standardized metagenomes accurately capture the qualitative (which dataset is higher/lower) and quantitative (how much higher/lower) richness differences between metagenomes. Note that the estimated richness ratio is not highly accurate at very low Nonpareil coverage. On the contrary, sequencing effort standardization even inverts the qualitative differences in richness showing higher diversity of Uneven100sp respect to Uneven1000sp when standardized to 1 million reads and generally shows much less accuracy.

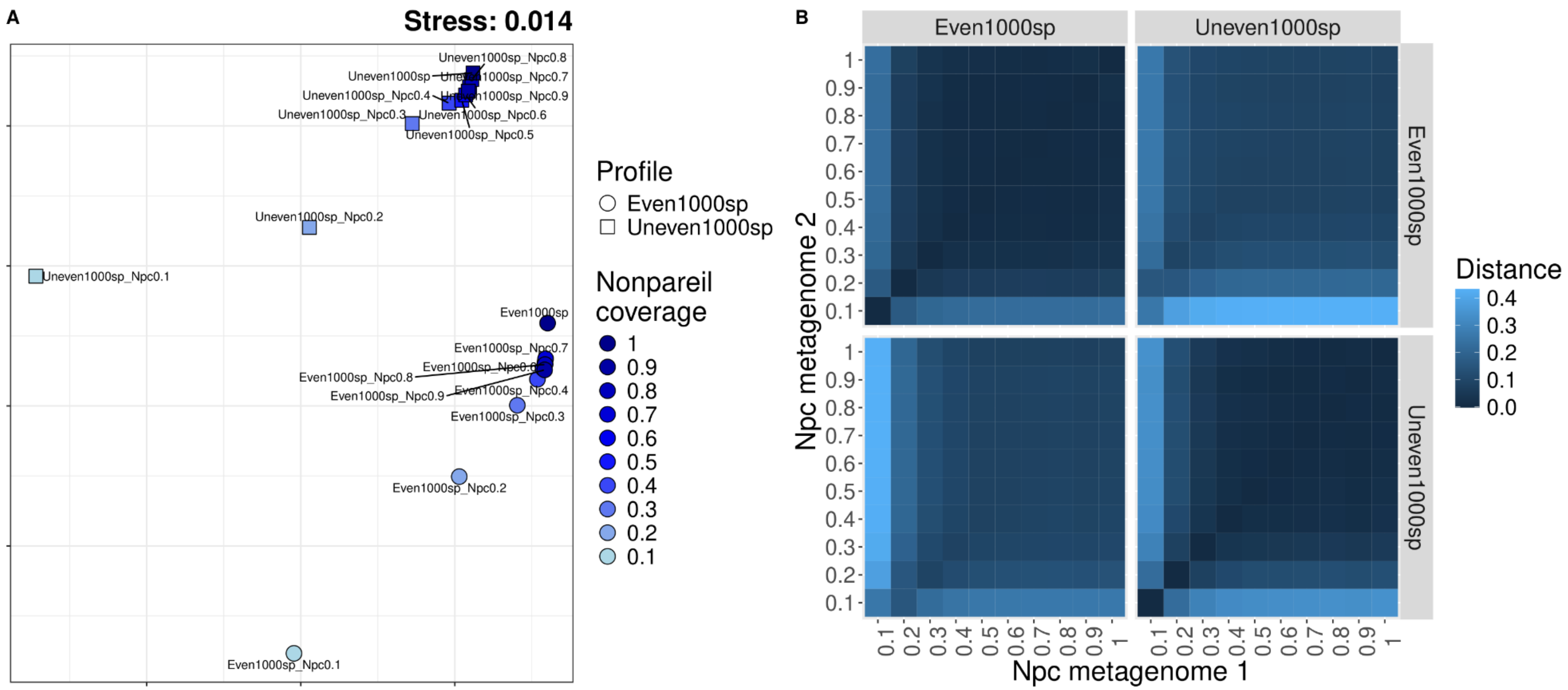

Figure S6. Impact of Nonpareil coverage on beta-diversity analyses. A) Non-metric multidimensional analysis (NMDS) based on the Bray-Curtis distances calculated using the relative abundance of genomes detected in Even1000sp (dots) and Uneven1000sp (squares) metagenomes subsampled at Nonpareil coverage values ranging from 1 (dark blue) to 0.1 (light blue). B) Heatmap showing the Bray-Curtis distances between metagenomes subsampled at varying Npc values. Note the increasing divergence of subsampled metagenomes as Nonpareil coverage decreases. Particularly, note the separation of metagenomes at Npc 0.3 and below from the original metagenome.

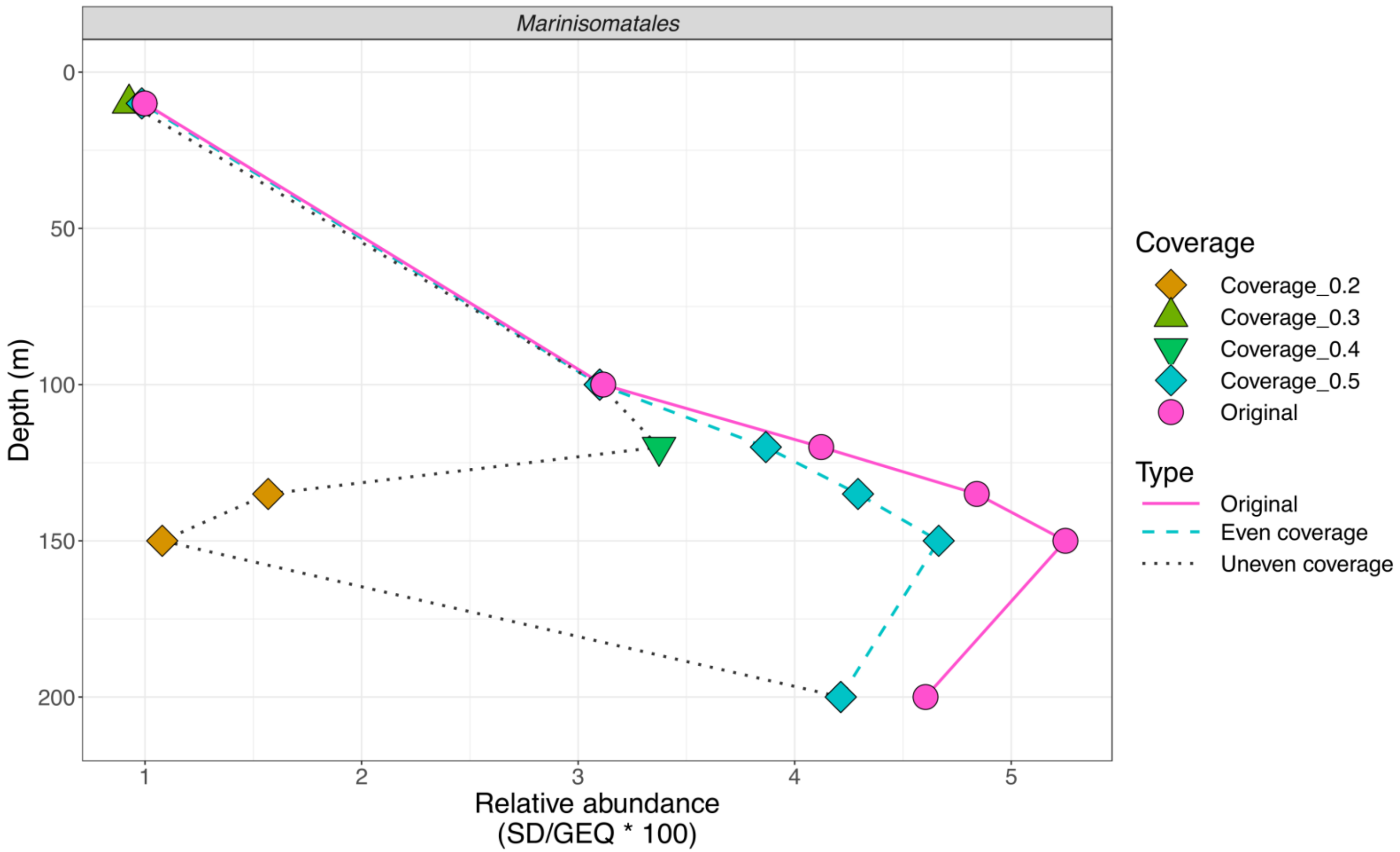

Figure S7. Comparison of abundance trends across depth in the original (full-size) metagenomes (solid line, average Npc=0.8) and subsampled metagenomes at the same (dashed line) and different Nonpareil (dotted line) coverage levels. The plot represents the aggregated relative abundance (x-axis) of MAGs belonging to the order *Marinisomatales* along the depth profile of the ocean (y-axis) based on the metagenomes provided by Hawley and colleagues. The points for the non-standardized (uneven) metagenomes were randomly selected from the data, simulating a potential scenario in which these metagenomes are analyzed together. Note the substantially different trends in the Npc standardized (even) vs. the non-standardized (uneven coverage) metagenomes.

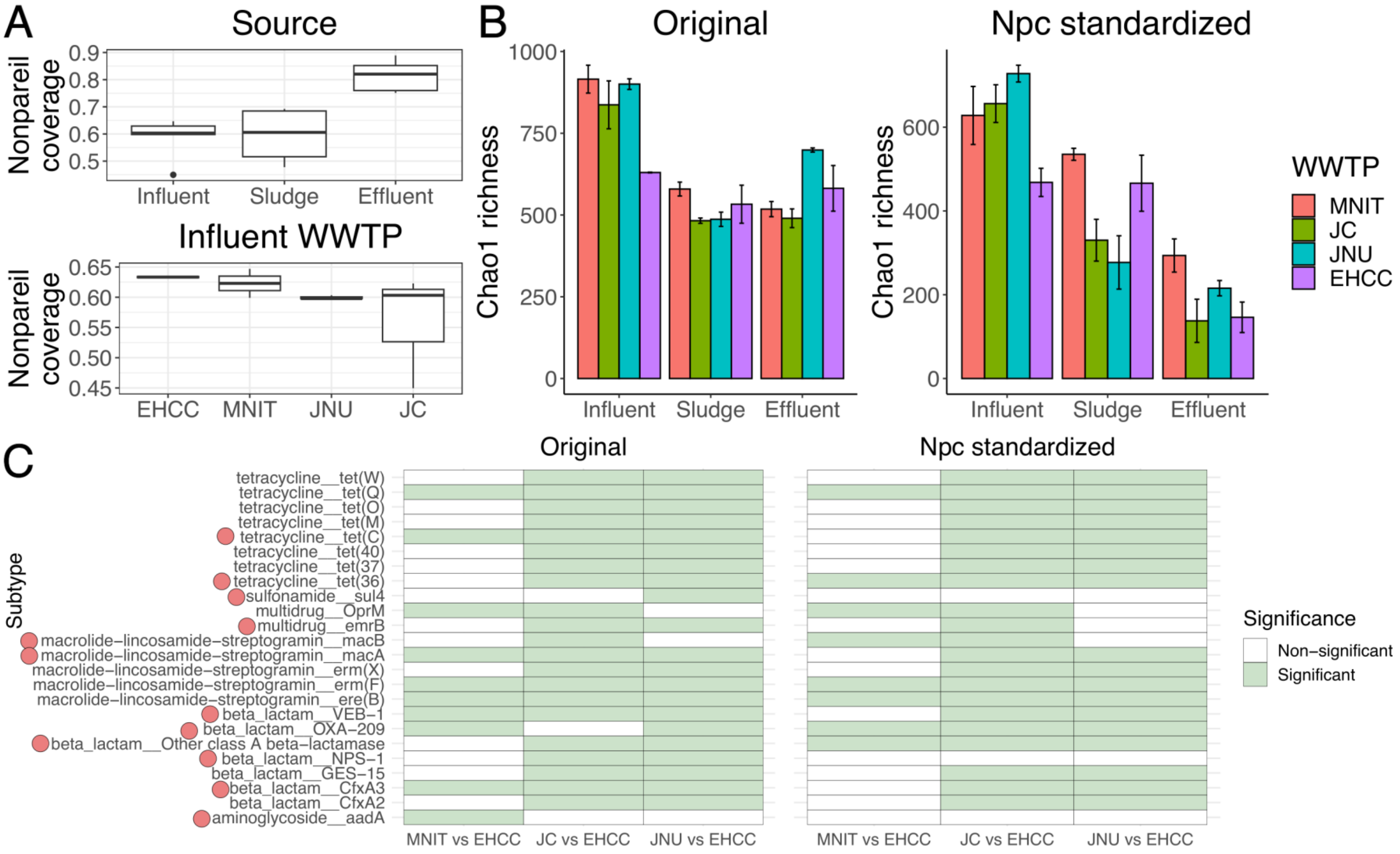

Figure S8. Reanalysis of the data by Zhang and colleagues (2021) showcases the effect of uneven coverage on inferring differential abundance of antibiotic resistance gene (ARG) subtypes. A) Nonpareil coverage (y-axis) of wastewater metagenomes collected from various stages (influent, sludge and effluent; x-axis top panel) of four different wastewater treatment plants (WWTP; x-axis bottom panel). Note the higher Nonpareil coverage of effluent metagenomes compared to influent and sludge metagenomes as well as the different

nonpareil coverage for each WWTP. B) Chao1 richness (y-axis) of ARGs in influent, sludge and effluent metagenomes (x-axis) of the four WWTP (colors) calculated for the original metagenomes and the Nonpareil coverage standardized metagenomes (Npc=0.45, the lowest Npc in the dataset). Error bars show the standard deviation of the 3 replicates per source and WWTP (influents from MNIT and EHCC had 2 replicates). Note that ARGs richness between sludge and effluents was similar in the original metagenomes but effluents showed significantly lower richness after correcting by Nonpareil coverage. Richness of ARGs in the effluent samples was thus overestimated by the original study due to higher coverage of the latter samples (see A). C) Differential abundance analysis of ARGs subtypes (y-axis) in the original and the Nonpareil coverage standardized metagenomes obtained from influent samples. The EHCC WWTP was compared to the three other WWTP (MNIT, JC and JNU). Green cells indicate statistically significant differences (p-value < 0.05) using the Welch's test while white cells indicate no statistically significant differences. The subtypes shown here correspond to the same subtypes that were shown as statistically significant in the original publication (some ARG subtypes could not be analyzed here due to the different version of the ARG-OAP database used). ARG subtypes that displayed a distinct result between the original and the Nonpareil coverage standardized metagenomes are highlighted with pink circles next to the name. Note that the results were different for about half of the ARG subtypes, indicating that uneven coverage could often lead to misleading results.

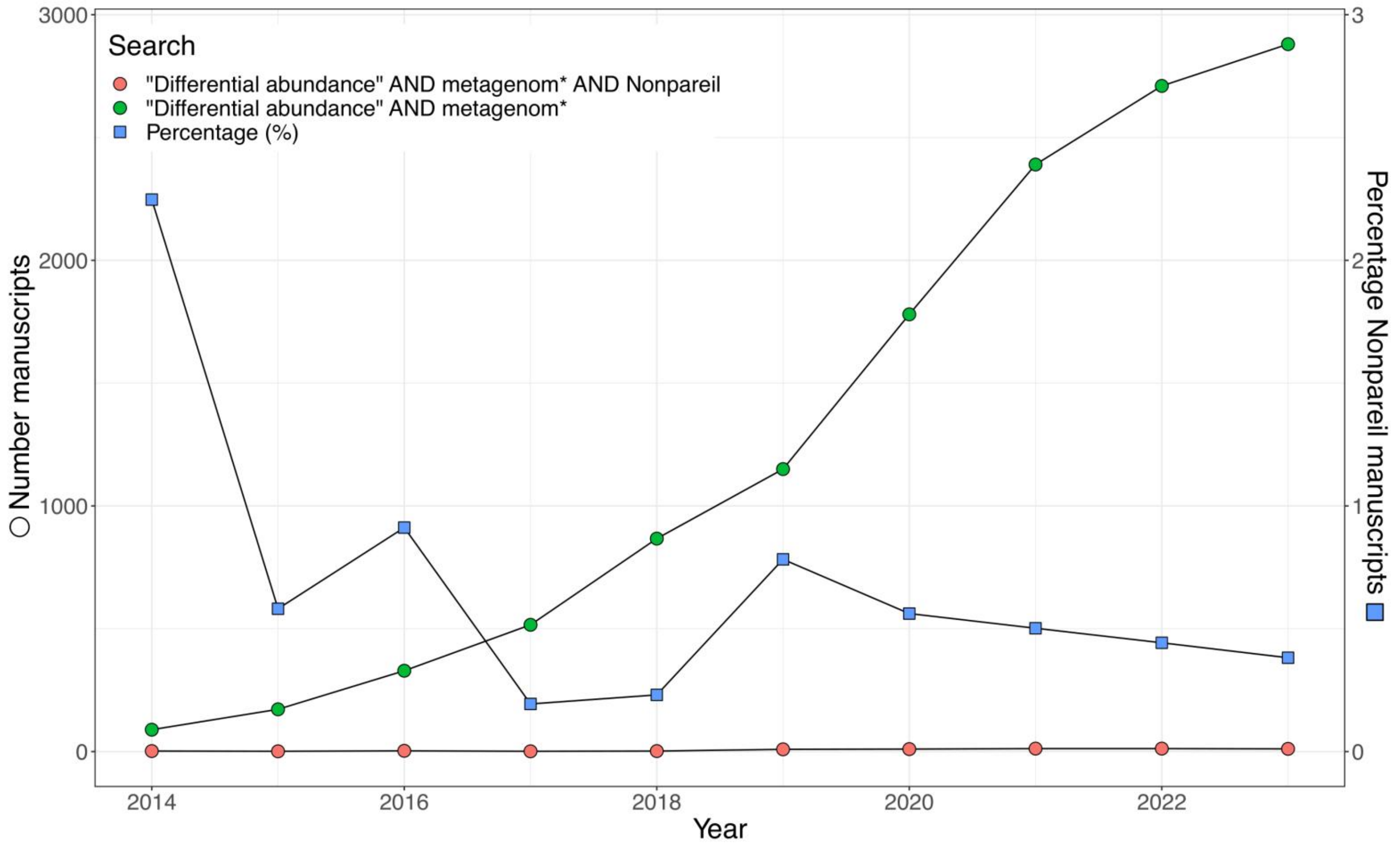

Figure S9. Number of manuscripts (y-axis) published in the last 10 years (x-axis) that mentioned metagenomic differential abundance analysis, including how many also mentioned the Nonpareil tool. The search was performed on Google Scholar on August 8th 2024, using the strings shown in the legend. Dots represent the number of manuscripts that, in addition to differential abundance and metagenomics, also mentioned (red) or not (green) Nonpareil. These values were used to calculate the percentage of manuscripts (blue squares; secondary y-axis) that mentioned Nonpareil from the total number of manuscripts that mentioned metagenomic differential abundance analysis. Note that Nonpareil was not mentioned in more than 99% of the manuscripts.

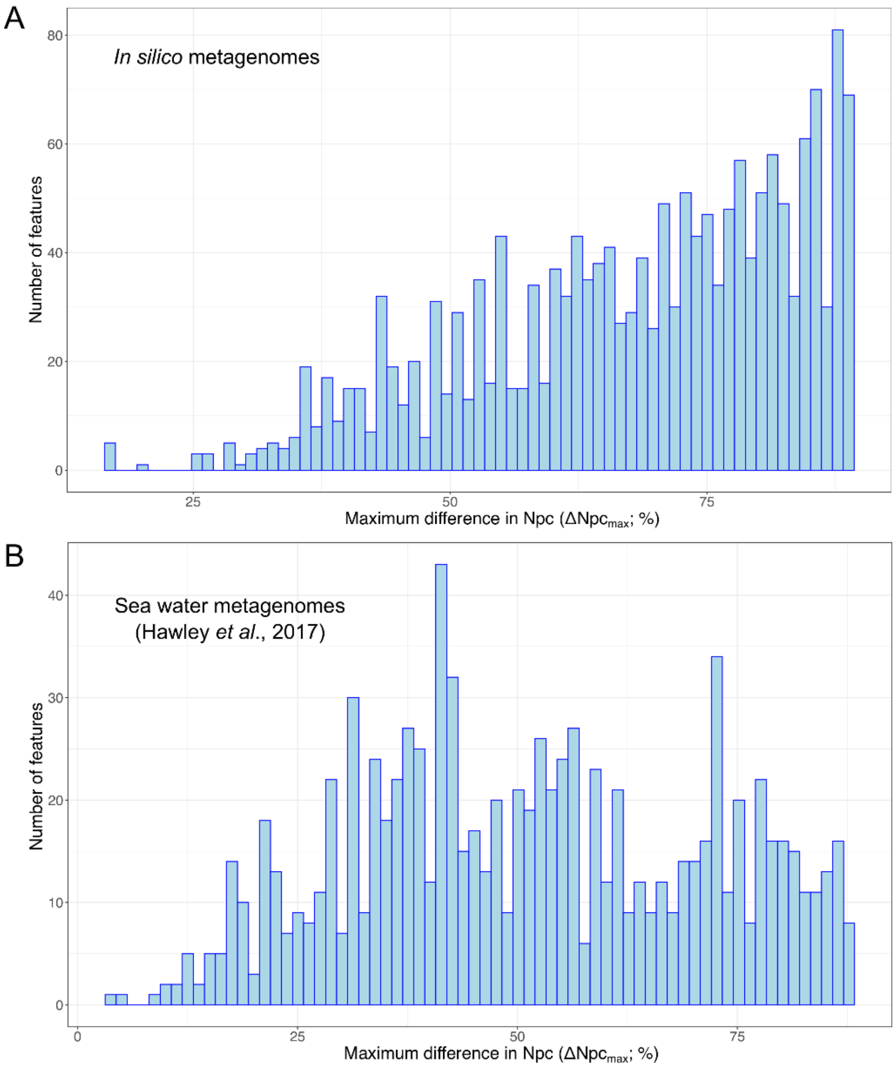

Figure S10. Histogram of the maximum difference in Nonpareil coverage (ΔNpc$_{max}$) between subsampled metagenomes that provided unbiased estimations of relative abundance differences compared to the full metagenomes. The histograms obtained with the *in silico* metagenomes (A) and real sea water metagenomes (B) are shown. A feature represents a group of MAGs belonging to the same taxonomic group: Phylum, Class, Order or Family). ΔNpc$_{max}$ values indicates the maximum difference in Npc that provided unbiased abundance results in subsampled metagenomes relative to the full metagenome. The histograms show the number of features (y-axis) plotted against their corresponding ΔNpc$_{max}$ value (x-axis). A total of 860 and 1,289 features were analyzed for the *in silico* and sea water metagenomes, respectively. Note the wide distribution obtained with not a clear threshold in ΔNpc$_{max}$. See the Methods section for details of the analysis performed to calculate ΔNpc$_{max}$ and main text for additional description of the results obtained.

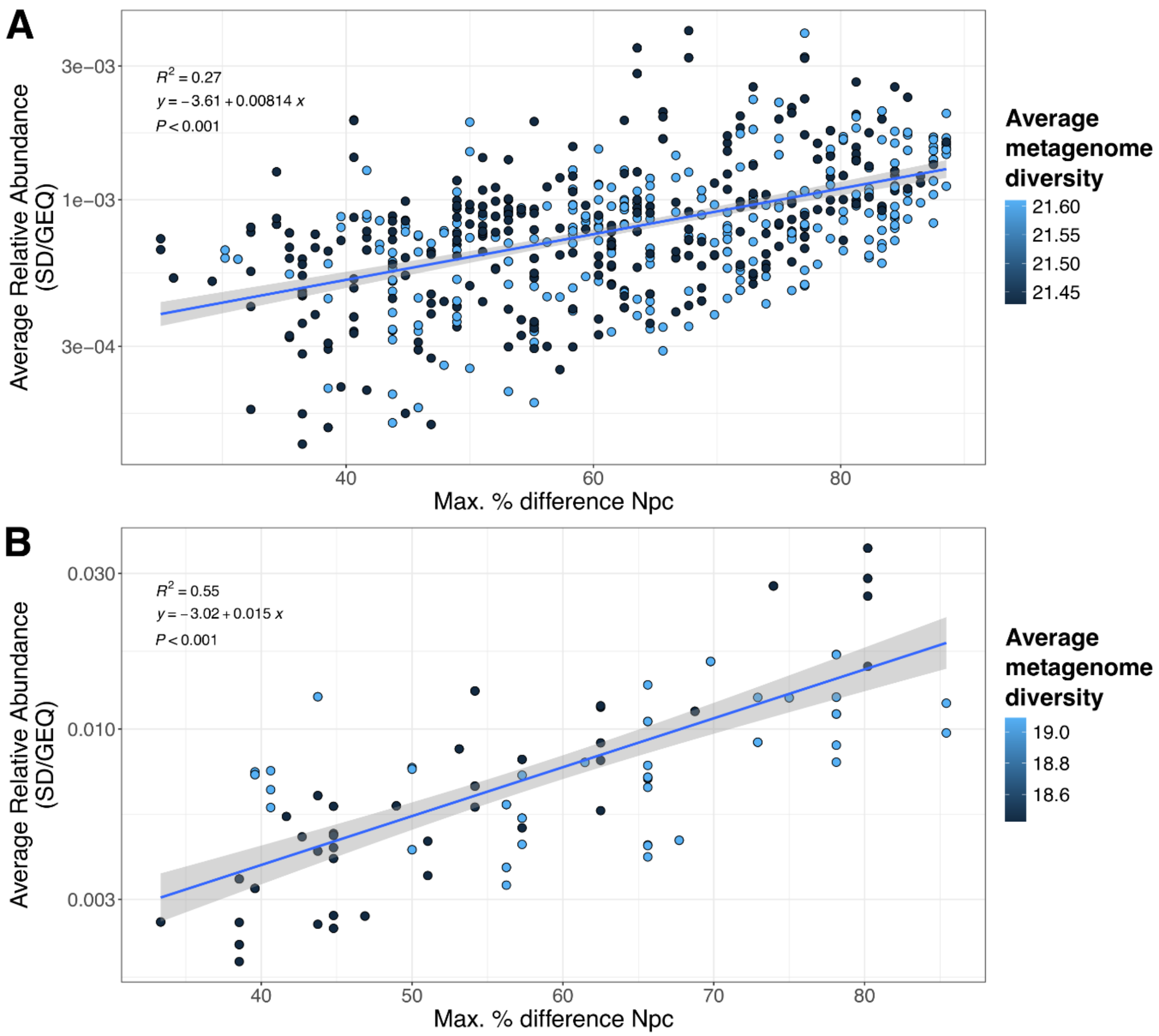

Figure S11. Relationship between average relative abundance (x-axis) and maximum Npc difference (ΔNpc_{max}) for unbiased results (y-axis) using the *in silico* metagenomes with 1,000 (A) and 100 species (B). Each dot represents one feature, which denotes a group of MAGs belonging to the same taxonomic group (Phylum, Class, Order or Family). The dot represents the average aggregated abundance of the members of the feature (x-axis) in a group of metagenomes (e.g., metagenomes with the same richness and evenness) relative to the maximum acceptable difference in Npc (ΔNpc_{max} in %; y-axis) to obtain the same abundance estimate as in the original metagenomes (meaning statistically unsignificant by Welch's test). ΔNpc_{max} is expressed as a fraction (percentage) of the Npc value of the original, non-subsampled metagenome. Colors in dots represent the average Nonpareil diversity for the group of metagenomes used in the analysis. Note the significant (p-value < 0.001) and positive correlation obtained in both cases

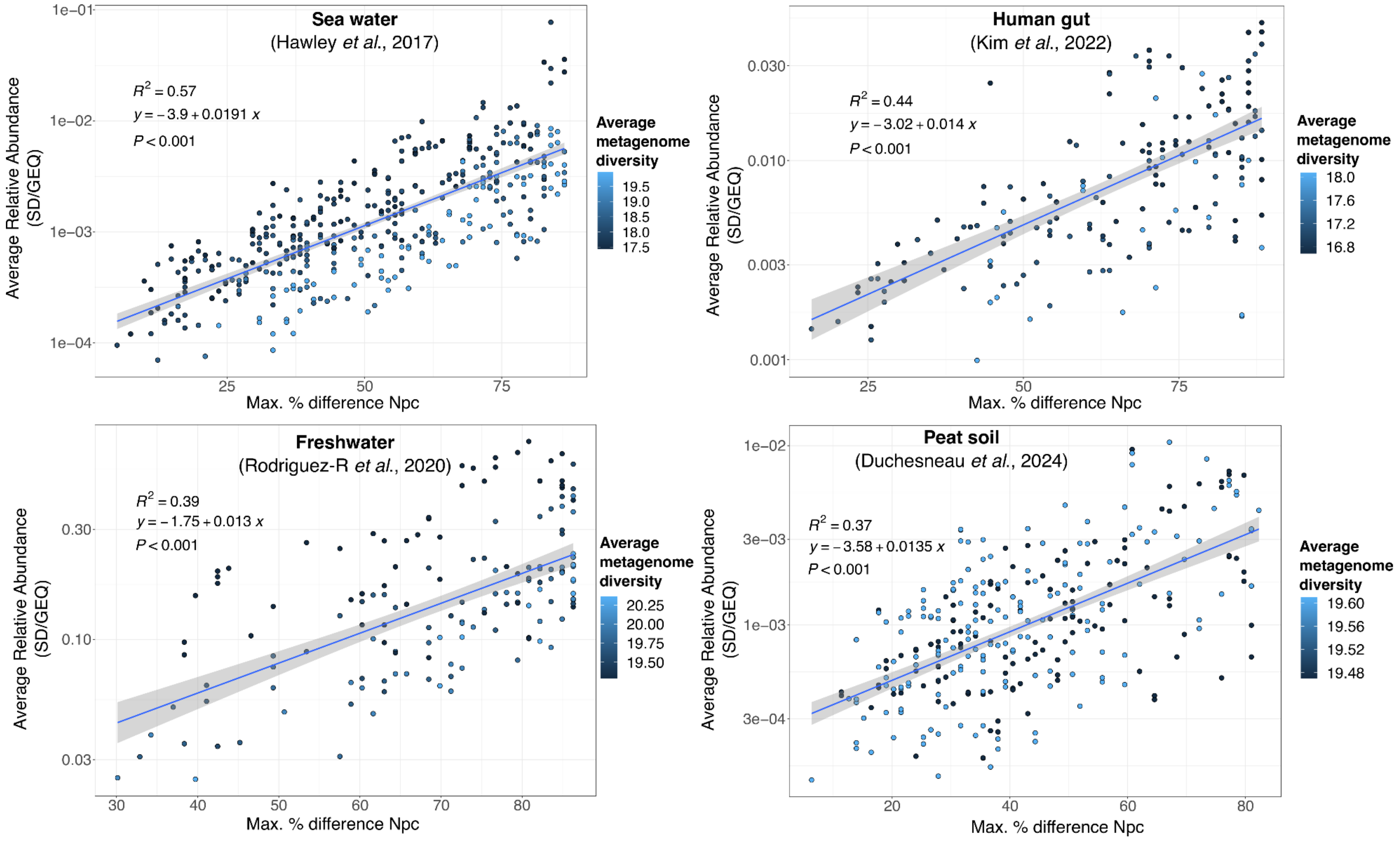

Figure S12. Relationship between average relative abundance (x-axis) and maximum Npc difference for unbiased results (y-axis) using real natural metagenomes. Each dot represents one feature, which represents a group of MAGs belonging to the same taxonomic group (Phylum, Class, Order or Family). The dot represents the average aggregated abundance of the members of the feature (x-axis) in a group of metagenomes (e.g., metagenomes from the same depth in A or same lake in C) relative to the maximum acceptable difference in

Npc (ΔNpc_{max} in %; y-axis) to obtain the same abundance estimate as in the original metagenomes (meaning statistically unsignificant by Welch's test). ΔNpc_{max} is expressed as a fraction (percentage) of the Npc value of the original, non-subsampled metagenome. The analysis was performed with four different environments: seawater (A), human gut (B), freshwater (C) and peat soils (D) to cover a wide range of habitats and Nonpareil diversity values (from 16.8 to 20.25). Colors in dots represent the average Nonpareil diversity for the group of metagenomes used in the analysis. Note that the positive correlation was strong and consistent for all four environments. The equation provided in each plot can be used to calculate the maximum acceptable difference in Npc (in %) for performing abundance comparison between two metagenomes (or their subsamples) based on a given relative abundance value of a target feature.

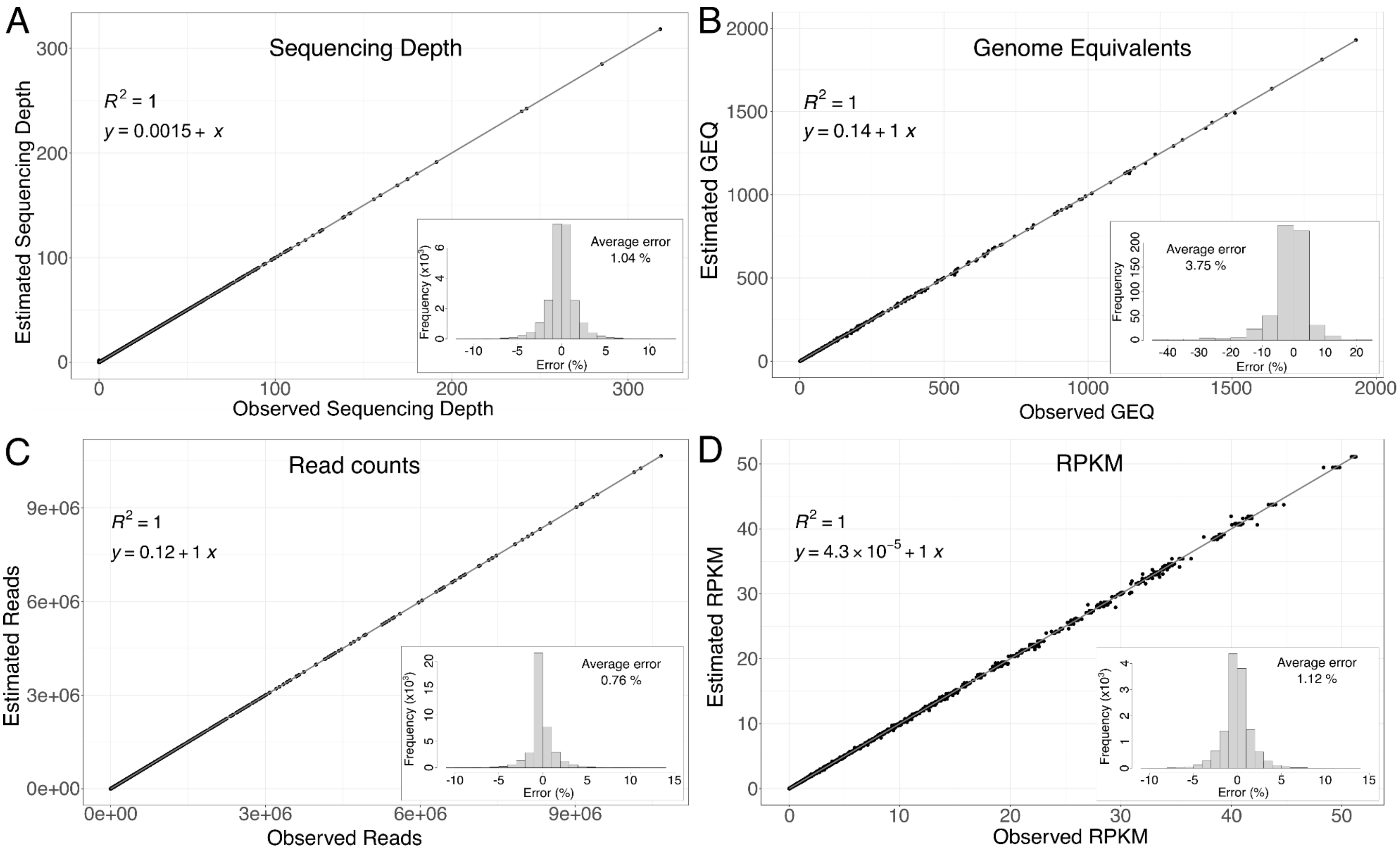

Figure S13. Accuracy of the relative abundance and read count estimation in subsampled metagenomes obtained with the Npc_standardization.R script. The accuracy was assessed for several metrics, namely Sequencing Depth (panel A), Genome Equivalents (GEQ; panel B), read counts (panel C) and RPKM (panel D). For each metric, dots represent the estimated value of each (group of) MAG(s) (panels A, C and D) or metagenome (for GEQ in panel B) calculated by the script (y-axis) compared to the actual observed value (x-axis)

obtained by manually subsampling and mapping the subsampled metagenomes to the same reference MAG dataset. The estimated values for the four metrics obtained by the script show an almost perfect correlation against the observed values (R^2=1) and very low percentages of error (~1%; bar plots at the top left of each panel). The marine metagenomes obtained by Hawley and colleagues was used for these tests.
